# Functional Evolution of Subolesin/Akirin

**DOI:** 10.3389/fphys.2018.01612

**Published:** 2018-11-13

**Authors:** Sara Artigas-Jerónimo, Margarita Villar, Alejandro Cabezas-Cruz, James J. Valdés, Agustín Estrada-Peña, Pilar Alberdi, José de la Fuente

**Affiliations:** ^1^SaBio, Instituto de Investigación en Recursos Cinegéticos (IREC), CSIC, Universidad de Castilla-La Mancha (UCLM), Junta de Comunidades de Castilla – La Mancha (JCCM), Ciudad Real, Spain; ^2^UMR BIPAR, INRA, ANSES, Ecole Nationale Vétérinaire d’Alfort, Université Paris-Est, Paris, France; ^3^Faculty of Science, University of South Bohemia, České Budějovice, Czechia; ^4^Institute of Parasitology, Biology Centre, Czech Academy of Sciences, České Budějovice, Czechia; ^5^Department of Virology, Veterinary Research Institute, Brno, Czechia; ^6^Facultad de Veterinaria, Universidad de Zaragoza, Zaragoza, Spain; ^7^Department of Veterinary Pathobiology, Center for Veterinary Health Sciences, Oklahoma State University, Stillwater, OK, United States

**Keywords:** immune response, vaccine, interactome, regulome, phylogeny, tick, *Anaplasma phagocytophilum*

## Abstract

The Subolesin/Akirin constitutes a good model for the study of functional evolution because these proteins have been conserved throughout the metazoan and play a role in the regulation of different biological processes. Here, we investigated the evolutionary history of Subolesin/Akirin with recent results on their structure, protein-protein interactions and function in different species to provide insights into the functional evolution of these regulatory proteins, and their potential as vaccine antigens for the control of ectoparasite infestations and pathogen infection. The results suggest that Subolesin/Akirin evolved conserving not only its sequence and structure, but also its function and role in cell interactome and regulome in response to pathogen infection and other biological processes. This functional conservation provides a platform for further characterization of the function of these regulatory proteins, and how their evolution can meet species-specific demands. Furthermore, the conserved functional evolution of Subolesin/Akirin correlates with the protective capacity shown by these proteins in vaccine formulations for the control of different arthropod and pathogen species. These results encourage further research to characterize the structure and function of these proteins, and to develop new vaccine formulations by combining Subolesin/Akirin with interacting proteins for the control of multiple ectoparasite infestations and pathogen infection.

## Introduction

Akirin, from the Japanese “akiraka ni suru" meaning “making things clear," was first identified by Goto et al. (2008) as a key component of the immune deficiency (IMD) and Tumor necrosis factor (TNF)/Toll-like receptor (TLR)-nuclear factor-kappa B (NF-kB) (TNF/TLR) signaling pathways in *Drosophila melanogaster* and *Mus musculus*, respectively. However, previous reports identified *akirin* as a gene involved in developmental processes in flies (Peña-Rangel et al., 2002; DasGupta et al., 2005). Subolesin, from the Latin “suboles" meaning “progeny," was first reported in 2003 with its discovery as the candidate protective antigen 4D8 by expression library immunization in *Ixodes scapularis* (Almazán et al., 2003). Gene orthology is a key concept in functional evolution (Koonin, 2005). Orthologs genes, defined as derived from a single ancestral gene that diverged during speciation, usually perform equivalent or identical functions, while paralogs that originated after gene duplication are considered to have more divergent functions (Koonin, 2005; Adipietro et al., 2012). Studies at genome level have identified many orthologs genes between divergent species, but the functional equivalency of the proteins encoded by these genes has not been fully characterized (Koonin, 2005).

Subolesin/Akirin are encoded by orthologs evolutionarily conserved throughout the metazoan that play a role in the regulation of different biological processes including immune response (de la Fuente et al., 2006a; Beutler and Moresco, 2008; Goto et al., 2008; Galindo et al., 2009; Macqueen, 2009; Macqueen and Johnston, 2009; Wan and Lenardo, 2010). Two *akirin* paralogs encoding Akirin1 and Akirin2 have been identified in vertebrates, but the functional homolog for invertebrate Subolesin/Akirin appears to be Akirin2 ortholog (Beutler and Moresco, 2008; Goto et al., 2008; Galindo et al., 2009; Macqueen and Johnston, 2009).

Understanding the function of the cell interactome (protein-protein physical and functional interactions) and regulome (transcription factors-target genes interactions) in response to infection is critical toward a better understanding of host-pathogen interactions and the identification of potential targets for new interventions for the prevention and control of tick infestations and tick-borne diseases (Rioualen et al., 2017; de la Fuente, 2018). Subolesin/Akirin are involved in both cell interactome and regulome, and constitute a good model for the study of the functional evolution of these processes in response to infection. In this review, we integrated the evolutionary history of Subolesin/Akirin with recent results on their structure and function in different species to provide insights into the functional evolution of these regulatory proteins, and their potential as vaccine antigens for the control of ectoparasite infestations and pathogen infection.

## Evolution of Subolesin/Akirin

**FIGURE 1:**
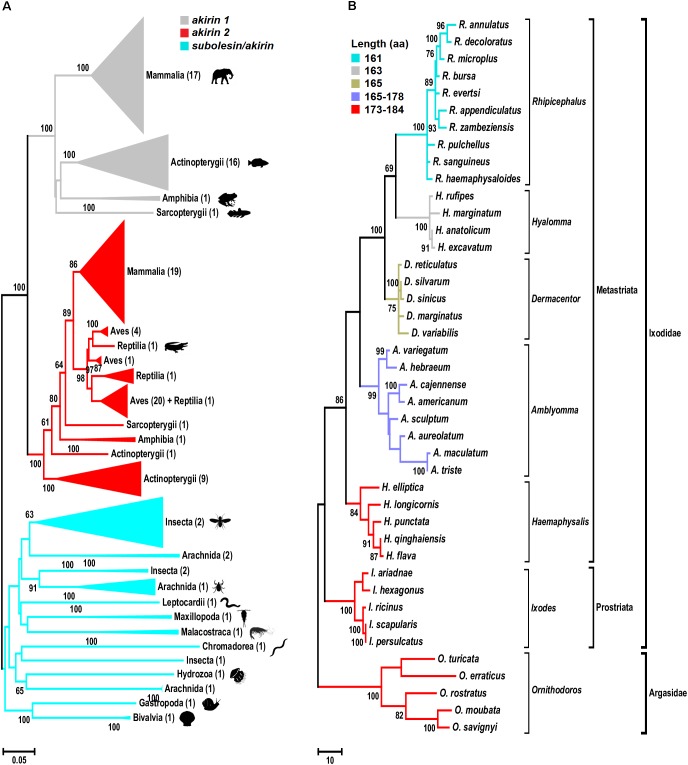
Phylogenetic analysis of *akirin* and *subolesin* nucleotide sequences. **(A)** A Neighbor Joining (NJ) phylogenetic tree was constructed with 361 nucleotide sequences belonging to 152 families, 73 orders and 15 classes (Mammalia, Actinopterygii, Amphibia, Sarcopterygii, Aves, Reptilia, Arachnida, Malacostraca, Insecta, Leptocardii, Maxillopoda, Chromadorea, Hydrozoa, Gastropoda and Bivalvia) of animals. All branches were collapsed at the class level and the number of orders per cluster is shown inside brackets. GenBank accession numbers and species names are provided in Supplementary Figure S1. Sequences were aligned using MAFFT configured for the maximum accuracy (Katoh and Standley, 2013). The final alignment contained 303 gap-free sites. All ambiguous positions were removed for each sequence pair. The best-fit model of the sequence evolution was selected based on Corrected Akaike Information Criterion (cAIC) and Bayesian Information Criterion (BIC) implemented in Molecular Evolutionary Genetics Analysis (MEGA) version 7. The Kimura 2-parameter model, which showed the lowest values of cAIC and BIC, was chosen for tree reconstruction. The evolutionary history was inferred using the NJ method implemented in MEGA 7 (Kumar et al., 2016). The percentage of replicate trees in which the associated taxa clustered together in the bootstrap test (500 replicates) is shown next to the branches (Felsenstein, 1985). **(B)** Phylogenetic tree of tick *subolesin* sequences. A Maximum Parsimony (MP) phylogenetic tree was constructed with 42 nucleotide sequences belonging to 6 and 1 genera of hard (family Ixodidae) and soft (family Argasidae) ticks, respectively. Because the evolution of *subolesin* in ticks has been less studied when compared to akirins, MP was used to generate a robust hypothesis on the evolution of this molecule in ticks. Sequences were aligned using MAFFT configured for the maximum accuracy (Katoh and Standley, 2013). Then, using the MAFFT alignment as template, a condon aligment was build (HIV database; www.hiv.lanl.gov accessed on 29-12-2017). The final alignment contained 576 total sites of which 329 were gap-free. The evolutionary history was inferred using the MP method (implemented in Molecular Evolutionary Genetics Analysis (MEGA) version 7 (Kumar et al., 2016). The percentage of replicate trees in which the associated taxa clustered together in the bootstrap test (500 replicates) is shown next to the branches (Felsenstein, 1985). The MP tree was obtained using the Subtree-Pruning-Regrafting (SPR) algorithm with search level 1 in which the initial trees were obtained by the random addition of sequences (10 replicates). Sequences were collected from Genbank and transcriptome projects and accession numbers are as follow: *Ixodes scapularis* (AY652654), *I. persulcatus* (KM888876), *I. ricinus* (JX193817), *I. ariadnae* (KM455971), *I. hexagonus* (JX193818), *Rhipicephalus evertsi* (JX193846), *R. appendiculatus* (DQ159967), *R. microplus* (EU301808), *R. sanguineus* (JX193845), *R. haemaphysaloides* (KP677498), *R. annulatus* (JX193844), *R. decoloratus* (JX193843), *R. zambeziensis* (GFPF01005851), *R. bursa* (GFZJ01017781), *R. pulchellus* (GACK01006228), *Dermacentor silvarum* (JX856138), *D. sinicus* (KM115649), *D. marginatus* (KU973622), *D. variabilis* (AY652657*), D. reticulatus* (JX193847), *Amblyomma variegatum* (JX193824), *A. hebraeum* (EU262598), *A. cajennense* (JX193823), *A. americanum* (JX193819), *A. maculatum* (JX193825), *A. aureolatum* (GFAC01005925), *A. triste* (GBBM01002796), *A. sculptum* (GFAA01000261), *Hyalomma anatolicum* (KT981976), *H. rufipes* (JX193849, *H. marginatum* (DQ159971), *H. excavatum* (GEFH01000904), *Haemaphysalis longicornis* (EU289292), *Hae. elliptica* (JX193850), *Hae. qinghaiensis* (EU326281, *Hae. flava* (KJ829652), *Hae. punctata* (DQ159972), *Ornithodoros moubata* (JX193852), *O. savignyi* (JX193851), *O. turicata* (GDIE01114362), *O. erraticus* (HM622148) and *O. rostratus* (GCJJ01005500).)

The phylogenetic analysis of *subolesin*/*akirin* coding sequences using an updated sequence database (Figure 1A and Supplementary Figure S1) expanded the information on the evolution of these genes, and supported the results reported previously by Macqueen and Johnston (2009) that *akirin1* and *akirin2* are vertebrate-specific paralogs that form a separate clade from invertebrate *subolesin*/*akirin*. In some vertebrate species, Akirins constitute a family of paralog proteins that probably originated as a result of whole-genome duplications (Macqueen and Johnston, 2009; Macqueen et al., 2010a,b; Liu et al., 2015). After *akirin* duplication, *akirin1* evolved faster than *akirin2*, the ortholog of tick *subolesin* (Figure 1A; Macqueen and Johnston, 2009). Furthermore, the loss of some *akirin* paralogs may have also occurred after genome duplications (Macqueen et al., 2010b; Liu et al., 2015). For example, the *subolesin/akirin* gene family consists of a single member in invertebrates (*subolesin/akirin*), birds and reptiles (*subolesin/akirin2*), two members in amphibians and mammals (*akirin1* and *akirin2*), two to three members in teleosts, and more than three members in Salmonidae (Macqueen et al., 2010a,b; Liu et al., 2015).

The phylogenetic analysis of *subolesin* gene sequences was performed in 42 species belonging to 6 and 1 genera of hard (family Ixodidae) and soft (family Argasidae) ticks, respectively. The analysis corroborated previous results showing a reductive evolution in protein length (de la Fuente et al., 2006a; Figure 1B). The Subolesin amino acid (aa) sequence evolved from 173 to 184 aa in *Ornithodoros, Ixodes* and *Haemaphysalis* spp. to 161 aa in *Rhipicephalus* spp. (Figure 1B). It is generally accepted that evolution proceeds toward greater complexity at both the organismal and genomic levels. However, numerous examples of reductive evolution of parasites and symbionts have been described to challenge this notion (Wolf and Koonin, 2013). Wolf and Koonin (2013) proposed that quantitatively, the evolution of genomes appears to be dominated by reduction and simplification, punctuated by episodes of complexification. The reductive evolution process has been particularly documented in genomes that replicate within the domain of a host genome (Andersson and Kurland, 1998; Driscoll et al., 2017), but it has also been proposed to be involved in the origin of bacteria from eukaryotes (Staley, 2017). In arthropods, reductive evolution has been implicated in the evolutionary origin of other proteins such as type IV classical cadherins (Sasaki et al., 2017).

The protein length is subjected to systematic variation that relates to the cellular context in which it functions (Wang et al., 2011). For growth rate-optimized cells, the reduction in protein length constitutes an advantage by increasing their mass-normalized kinetic efficiencies (Ehrenberg and Kurland, 1984; Kurland et al., 2007; Wang et al., 2011). Consequently, shorter proteins that retain maximum functional rates are expected to support faster cell growth rates than longer proteins with similar kinetic characteristics. Wang et al. (2011) proposed the use of the terms “domain” and “linker” to refer to protein folded domains and nondomain regions, respectively. Proteins with nondomain sequences are proteins intrinsically unstructured or natively unfolded that lack a stable tertiary structure but have a dynamic range of conformations (Orengo and Thornton, 2005). These proteins appear to be more abundant in eukaryotes when compared to prokaryotes and are usually involved in binding and molecular recognition (Brown et al., 2011). Subolesin/Akirin were characterized as a linker with three predicted disordered nondomain regions that resulted in unstructured proteins (Prudencio et al., 2010; see also below). These results agreed with the findings of Wang et al. (2011) that the evolutionary reductive constraints on protein lengths are preferentially expressed in linker sequences.

It is difficult to establish a comprehensive record of ticks developmental rates because most of the experiments in previously published papers have been done at different regimes of temperature, relative humidity and photoperiod, all factors affecting the time in which ticks complete each developmental stage. However, data compiled by Hoogstraal (1956), Morel (2003) and Horak et al. (2018) under similar conditions established that in the range of 24–28°C, ticks of the genera *Hyalomma* and *Rhipicephalus* complete their life cycle in about 33% less time than ticks of the genera *Ixodes* or *Amblyomma*. The *Hyalomma* and *Rhipicephalus* spp. are considered the two most recent genera of ticks, while *Ixodes* spp. and *Amblyomma* are among the most ancient splits of tick lineages (Mans et al., 2016; Figure 1B). Therefore, it is possible that the reductive evolution of Subolesin is associated with faster developmental rates in *Rhipicephalus* and *Hyalomma* spp. when compared to more ancient tick species even if they are sympatric. The faster developmental rate in recently evolved tick species may be associated with increasing cell growth rates that have been associated with reductive evolution (Wang et al., 2011). However, the complete association between existing data about developmental rates and evolutionary features of ticks requires further research.

**FIGURE 2:**
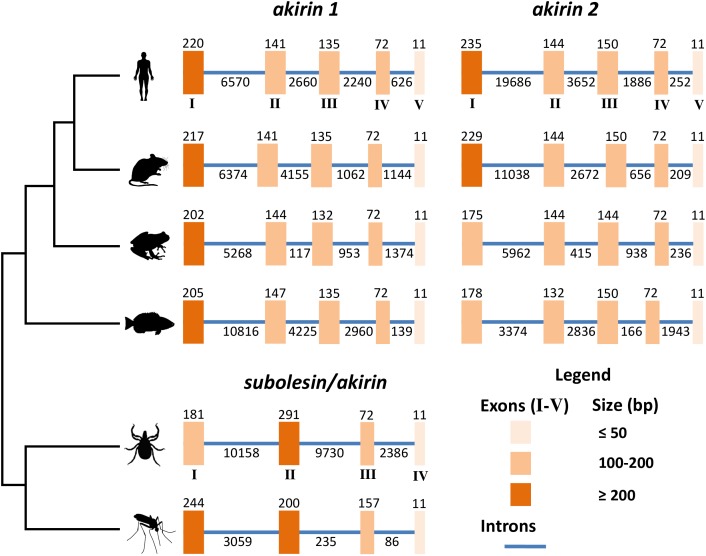
Genomic organization of *subolesin/akirin* orthologs across selected eukaryotic species. The genomic organization of the coding regions of tick (*I. scapularis*), human (*Homo sapiens*), mouse (*M. musculus*), frog (*Xenopus laevis*), fish (*Danio rerio*) and mosquito (*Anopheles gambiae*) *subolesin/akirin* is shown. The genomic organization of human, mouse, frog and fish *akirins* was previously reported (Liu et al., 2015). The genomic organization of tick and mosquito *subolesin/akirin* was collected from VectorBase (https://www.vectorbase.org; Giraldo-Calderón et al., 2015). Latin numerals correspond to the size of exons/introns in base pairs.

At the genome level, *subolesin*/*akirin* exon-intron architecture shows a clear evolutionary pattern (Figure 2). As shown for *subolesin*/*akirin* coding sequences (Figure 1A), the vertebrate-specific paralogs form a separate clade from invertebrate genes (Figure 2). The *subolesin*/*akirin* genes evolved from 4 exons in invertebrates to 5 exons in vertebrates. The exon sizes showed a pattern of larger to shorter for vertebrate *akirin1* exons I to V and mosquito *akirin* exons I to IV (Figure 2). However, for tick *subolesin* and vertebrate *akirin2* genes, the size of exons II and III, respectively, were larger or similar than that of preceding exon (Figure 2). Furthermore, while the length of 11 nucleotides (nt) in the last exon was conserved across evolution, the length of the penultimate exon evolved from 157 nt in mosquitoes to 72 nt in ticks and vertebrates (Figure 2). However, the length of the exon-intron sequence increased from 3,992 nt in mosquitoes to 22,829 nt in ticks and then decreased to 8,862 nt in fish to increase back again to 26,088 nt in humans (Figure 2). These results do not correlate with genome sizes of these organisms (Gregory, 2005), and may originated from still unknown evolutionary events.

## Subolesin/Akirin Structure and Its Interactions With Dna and Transcription Factors

**FIGURE 3:**
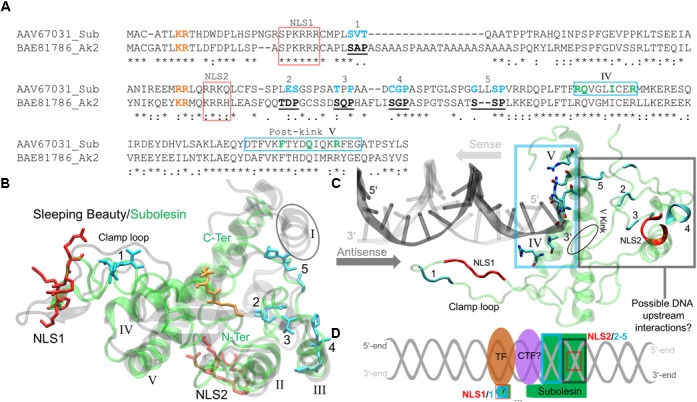
The *I. scapularis* Subolesin structure and its interactions with DNA and transcription factors. **(A)** The pairwise sequence alignment of the *I. scapularis* Subolesin (Sub) and the rat Arkinin2 (Ak2), accession numbers indicated, was generated using the MAFFT alignment program at default settings (Katoh et al., 2017). The NLS 1 and 2 domains (red box), binding sites 1-5 (bold-underlined in Ak2 and cyan for Sub), and the novel DNA binding sites (green and enclosed in a cyan box) are shown. The residues color-coded orange are extensions of the NLS domains. **(B)** The superposed tertiary structures of Sleeping Beauty (transparent black) and Subolesin (transparent green) are represented with the clamp loop labeled and the five α-helices of Sleeping Beauty (PDB: 5CR4) annotated in roman numerals. The tertiary residue positions of the labeled Subolesin NLS domains and binding sites are, respectively, color-coded as in the pairwise alignment. The Subolesin termini positions are color-labeled (green). **(C)** The Subolesin-DNA complex, modeled from the Mos1-DNA (PDB: 3HOS) show the residues of the novel DNA-binding site on α-helices IV-V, enclosed by a cyan box that were predicted by I-TASSER (Zhang, 2008). The DNA prime ends are color-labeled for the respective directions (indicated by arrows) of the sense (gray) and antisense (dark gray) strands. The residue positions of the Subolesin clamp loop, NLS domains and binding sites are color-coded as in previous panels A and B. **(D)** The schematic representation of the upstream DNA (gray helix) interactions with Subolesin NLS2, binding sites 2-5, and the potential clamp loop interaction (via NLS1 and binding site 1) with an unknown co-transcription factor (CTF?) and unknown (?) transcription factor (TF).

Akirins are involved in cellular processes that are regulated by specific domains and binding sites. The rat Akirin2 (or 14-3-3*β* Interactant 1) described by Komiya et al. (2008) is 48% identical to the *I. scapularis* Subolesin and was previously used to identify the conserved Akirin nuclear localization signal (NLS) domains and its binding sites (Macqueen and Johnston, 2009). The pairwise sequence alignment shows that both NLS domains of the rat Akirin2 are conserved in Subolesin, but with variations in a few binding sites (Figure 3A). Subolesin binding sites 1 and 4 are similar to Akirin2 with Subolesin binding sites 2 and 3 each possessing a single substitution. The Subolesin binding site 5, however, has a double Leu insertion when compared to Akirin2 (Figure 3A). Currently there are no resolved Subolesin/Akirin structures and standard sequence-based bioinformatics methods lack parameters for locating structural homologs in the Protein Databank (PDB). Therefore, several logical steps were taken to correctly model the *I. scapularis* Subolesin. The Subolesin sequence (Figure 3A) was initially submitted to I-TASSER (Zhang, 2008), a protein multiple threading algorithm that is considered a top competitor in the Critical Assessment of Structure Prediction^1^. The I-TASSER algorithm resulted in five distinct Subolesin models that were then individually submitted to the DALI server (Holm and Laakso, 2016) for identifying PDB structural homologs of similar length with minimalα-carbon backbone deviations between the two global structures. Since Subolesin/Akirin are effectors of the IMD/TNF/TLR Relish/NF-kB signaling pathways (Goto et al., 2008; Naranjo et al., 2013), a match with homologous structures that potentially participate within these pathways was also a criterion in selecting an appropriate Subolesin model. This logical approach resulted in a Subolesin model homologous in structure, with only 7% residue conservation, to the genetically engineered catalytic domain of the transposase Sleeping Beauty (Ivics et al., 1997; Zanesi et al., 2013; Figure 3B).

As part of the *Tc1/mariner* transposon superfamily, the Sleeping Beauty transposase has been engineered for genetic screening studies, leading to the discovery of several genes activated by Sleeping Beauty transposon insertions that participate in the NF-kB signaling pathway (Zanesi et al., 2013). The Sleeping Beauty transposase sequence is composed of an N-terminus paired-like domain with a leucine zipper (∼90 residues long) and the C-terminus folds as the catalytic domain (Ivics et al., 1997). The catalytic domain of Sleeping Beauty was resolved with a DNA transposon end and modeled with a target DNA revealing the mechanism of hyperactive Sleeping Beauty mutation screening studies while discovering novel variants for future screenings (Voigt et al., 2016). The Sleeping Beauty crystal structure details that its catalytic domain has a global homology to Ribonuclease H (RNase H) (Voigt et al., 2016). The RNase H-like protein fold forms a catalytic triad (Asp-Asp-Glu) that coordinates metal ions involved in excision and insertions of DNA (Voigt et al., 2016). By resolving the Sleeping Beauty catalytic domain, Voigt et al. (2016) also discovered that the Gly-rich box (located on the clamp loop) is involved in protein-protein interactions, specifically with partnering monomers in the DNA complex. The conserved positions of the catalytic triad and the Gly-rich box, however, are not present in Subolesin/Akirin sequences.

Prior to acting in the *Tc1/mariner* transposon system, Sleeping Beauty must enter the nucleus. Passage to the nucleus is controlled by NLS domains that have a strong affinity to karyopherin/importin receptors, proteins responsible for transporting NLS-tagged “cargo” in and out of the nucleus via nuclear pores (Leung et al., 2003). The N-terminus of the Sleeping Beauty catalytic domain contains a NLS domain that is quite long (17 residues) and is actually a bipartite NLS (Ivics et al., 1997). A monopartite NLS domain has the formulation Lys-Lys/Arg-[X]-Lys-Lys/Arg, where [X] is any other (∼2) amino acids. A bipartite NLS domain has a linker sequence, where [X] is ∼10 residues long (Makkerh et al., 1996). Mutations of these upstream NLS residues prior to the linker sequence [X] has shown to reduce protein entry into the nucleus (Dingwall et al., 1988), and inhibit NLS binding to karyopherin/importin receptors (Leung et al., 2003). These upstream, positively charged residue pairs are in the aligned sequences of Figure 3A (orange-labeled residues), indicating that Subolesin/Akirin2 NLS1 is bipartite. The two positively charged residues highlighted near NLS2 (Figure 3A) indicate that the NLS2 of Subolesin/Akirin is actually a longer monopartite domain. The modeled Subolesin structure has its NLS1 positioned on the clamp loop, which differs from Sleeping Beauty that is coordinated at the N-terminus α-helix (I) (Figure 3B). The Subolesin NLS2 domain, not present in the catalytic domain of Sleeping Beauty, is located on an α-helix (II) outside the central core of the protein. The absence of the catalytic triad of Sleeping Beauty (Voigt et al., 2016) and metal binding sites in Subolesin/Akirin support that these proteins do not act as a transposase. Additionally, the Subolesin/Akirin binding sites have long been recognized by mutation studies (Komiya et al., 2008), and Subolesin/Akirin RNA interference (RNAi) experiments have shown to disrupt the Relish signaling pathway (Goto et al., 2008; Naranjo et al., 2013). However, as discussed bellow, the Subolesin/Akirin interactome has not been fully characterized, and whether Subolesin/Akirin binding partners are only proteins or also include nucleic acids.

The superposed structures of Subolesin and Sleeping Beauty depict a global homology with a low α-carbon backbone deviation of 0.3 nm (Figure 3B). There are, however, missing and disordered secondary structures. The Sleeping Beauty catalytic domain has five β-sheets surrounded by five α-helices. As previously mentioned, the N-terminus α-helix I of Sleeping Beauty that contains its NLS domain is missing in the Subolesin model (encircled in Figure 3B), thereby shifting the Subolesin NLS1 domain to the clamp loop. Moreover, the β-sheets of Subolesin are highly disordered. Future experiments should resolve the stacking and conformations of the disordered Subolesin β-sheets by X-ray crystallography. Nevertheless, the remaining four α-helices (II-V) are structurally conserved, and the Subolesin/Akirin binding sites 2-5 are positioned on or approximating α-helix III (Figure 3B). As in the primary sequence (Figure 3A), the Subolesin/Akirin binding site 1 is structurally distant from the other sites, located on the N-terminus clamp loop near the position of NLS1 (Figure 3B). As a transposase, the clamp loop of the resolved Sleeping Beauty catalytic domain is not in its DNA-bound conformation. Therefore, Voigt et al. (2016) modeled the clamp loop after the DNA-bound transposase, Mos1, from *Drosophila mauritiana* (Richardson et al., 2009). The Mos1 also has poor sequence identity to Subolesin (<5%), but are structurally homologous with α-carbon backbone deviation of 0.34 nm. This led to a subsequent I-TASSER simulation using the template Mos1 as conducted by Voigt et al. (2016). The resulting model has Subolesin bound to a DNA duplex with an adequate clamp loop conformation that extends downstream the duplex (Figure 3C).

The Subolesin-DNA complex show several residues on α-helices IV and V that approximate the DNA 5′-end of the sense strand and the 3′-end of the antisense strand (Figure 3C). Four of the seven residues positioned on α-helix IV mainly interact with the phosphate backbone of the antisense strand. The remaining three residues that interact with both strands are after the pivotal kink of α-helix V (encircled in Figure 3C). The alignment in Figure 3A highlights these novel DNA-binding residues and indicates the α-helix on which they are positioned. The binding sites 2–5 and NLS2 are distal to the DNA interacting site, while the clamp loop containing NLS1 and binding site one is downstream the DNA duplex. Given the structural coordination of Subolesin bound to DNA (Figure 3C), binding sites 2–5 and NLS2 may interact with nucleotides upstream the DNA or with additional co-transcription factors (CTF) (Figure 3D). The transcription factors (TF) Relish/NF-kB form DNA-protein complexes with CTFs, and Subolesin is hypothesized to act as a CTF of Relish via an intermediate CTF (Figure 3D and described in the next section). Furthermore, the extended DNA downstream position of the Subolesin clamp loop with binding site one will hypothetically coordinate the CTF and possibly the TF (Figure 3D). If Subolesin is a CTF, how will it conform while the mRNA is being transcribed? Positively charged residues, specifically Lys, recognize RNA strands via electrostatic interactions (Law et al., 2006). Given conformational flexibility of the Subolesin clamp loop and the fact that it does not contain the Sleeping Beauty Gly-rich box, the proximity of positively charged Subolesin NLS1 domain residues downstream the DNA duplex may guide transcribing mRNAs for post-transcriptional processing.

## Function of Subolesin/Akirin

**FIGURE 4:**
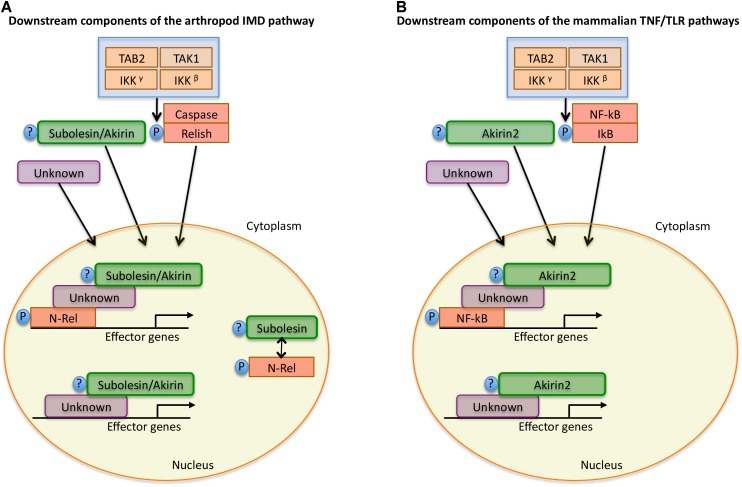
Model for Subolesin/Akirin function in immune response pathways. A simplified annotation of the downstream components of the arthropod IMD and mammalian TNF/TLR pathways (Goto et al., 2008; Beutler and Moresco, 2008; de la Fuente et al., 2008; Naranjo et al., 2013; Shaw et al., 2017). **(A)** After activation of the arthropod IMD pathway, the TGF-β (TAK1), Tak1-binding protein 2 (TAB2) and the I-KB kinase (IKK) complex are recruited, which leads to phosphorylation of the NF-κB transcription factor, Relish. After phosphorylation, the N-terminal domain of Relish (N-Rel) is cleaved by Caspase-8 homolog Dredd or a similar Caspase and is translocated to the nucleus. Subolesin/Akirin may be post-translationally modified and translocated to the nucleus. In the nucleus, N-Rel interacts with Subolesin/Akirin through unknown proteins to drive the production of anti-microbial peptides and other effector genes. In ticks, N-Rel and Subolesin may be reciprocally regulated. **(B)** In mammals, the activation of the TNF/TLR signaling pathways also results in the recruitment of the TAB2-TAK1 and IKK complexes, which results in the phosphorylation of the inhibitory regulator of NF-kB, IkB, resulting in the NF-kB translocation to the nucleus. As in arthropods, Akirin2 may be post-translationally modified and translocated to the nucleus. Once in the nucleus, NF-kB interacts with Akirin2 through unknown proteins for the activation of gene expression. In both arthropods and mammals, Subolesin/Akirin are involved in the regulation of genes that are Relish/NF-kB independent.

**FIGURE 5:**
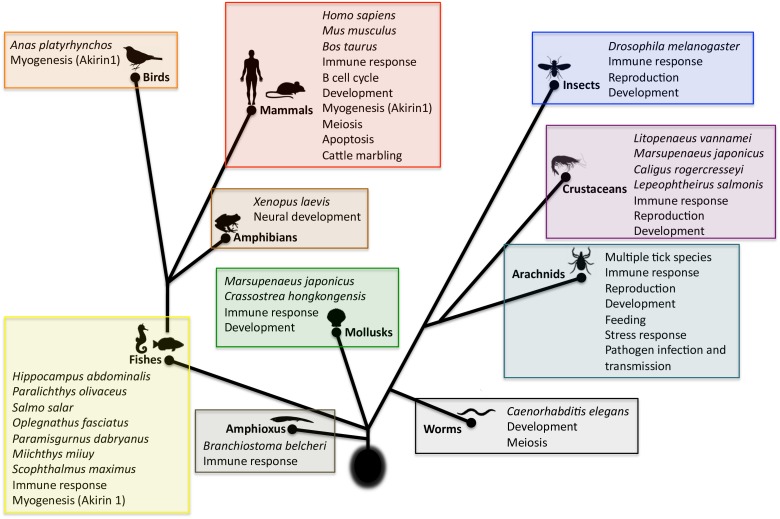
Scheme of the evolution and function of Subolesin/Akirin | Functional annotations were done based on published results for Subolesin/Akirin2. References are in the text of the paper. Myogenesis, attributed exclusively to Akirin1, was included and labeled as such. For each taxa, species in which studies were conducted are shown.

The innate immune response acts as the first line of defense against pathogen infection in all metazoans, and constitutes the only immune response in invertebrates (Medzhitov et al., 1997; Shaw et al., 2017). It has been shown that invertebrate Subolesin/Akirin and vertebrate Akirin2 act in concert with Relish/NF-kB to induce the expression of a subset of downstream pathway elements in the IMD and TNF/TLR signaling pathways involved in the immune response to pathogen infection. This function of Subolesin/Akirin has been documented in ticks (de la Fuente et al., 2008, 2017a; Naranjo et al., 2013; Gulia-Nuss et al., 2016; Shaw et al., 2017), fruit fly (Beutler and Moresco, 2008; Goto et al., 2008; Wan and Lenardo, 2010; Bonnay et al., 2014), human (Goto et al., 2008), salmon (Macqueen et al., 2010b), shrimp (Hou et al., 2013), Japanese flounder (Yang et al., 2013), amphioxus (Yan et al., 2013), rock bream (Kasthuri et al., 2013), Chinese loach (Xue et al., 2014), Hong Kong oyster (Qu et al., 2014), turbot (Yang et al., 2011), sea louse (Valenzuela-Muñoz and Gallardo-Escárate, 2014), mouse (Beutler and Moresco, 2008; Goto et al., 2008; Wan and Lenardo, 2010; Tartey et al., 2015), croaker (Liu et al., 2015); shrimp (Liu Y. et al., 2016; Liu et al., 2016), and seahorse (Pavithiran et al., 2018; Figures 4, 5).

**FIGURE 6:**
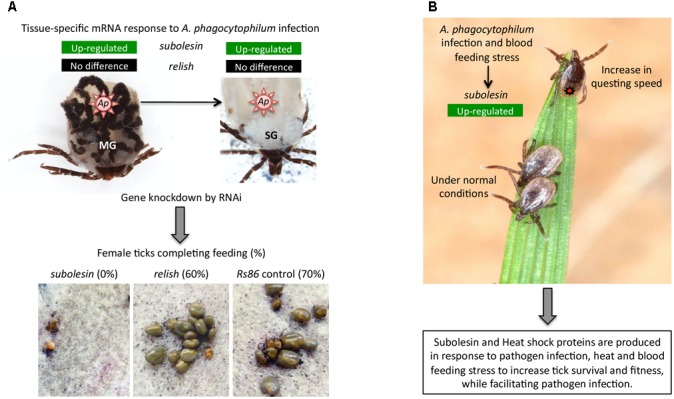
Examples of the role of tick Subolesin in different biological processes | **(A)** Role of tick Subolesin in *A. phagocytophilum* infection and blood feeding. The transcriptomics analysis in different *I. scapularis* tick tissues showed that *subolesin* (ISCW023283) but not *relish* (ISCW018935) mRNA levels significantly increased in response to *A. phagocytophilum* (*Ap*) infection in both midgut (MG) and salivary glands (SG). In addition, the *subolesin* gene knockdown phenotype in ticks injected with dsRNA resulted in a significant reduction in the number of female ticks completing feeding, oviposition and fertility. Results were reported by Ayllón et al. (2015). Photo of dissected *I. scapularis* partially fed adult female ticks courtesy of K. M. Kocan (Oklahoma State University, United States). **(B)** Role of tick Subolesin in *A. phagocytophilum* infection, blood feeding and questing speed. The response to *A. phagocytophilum* and stress increases *subolesin* levels, which together with heat shock proteins improve tick questing speed and survival. Results were reported by Busby et al. (2012). Photo of questing *Ixodes ricinus* courtesy of L. Grubhoffer & J. Erhard (Biology Center of the AS CR, Institute of Parasitology, Czechia).

Subolesin/Akirin are also involved in the Relish/NF-kB independent gene regulation (Figure 4), thus playing a role in various biological processes in addition to the immune response (Figure 5). These processes include animal reproduction and development, causing lethal embryonic or reduced growth phenotypes in knockout mice, fruit fly, ticks, and nematodes (Maeda et al., 2001; de la Fuente et al., 2006a; Goto et al., 2008; Carpio et al., 2013; Qu et al., 2014), metazoan myogenesis (Marshall et al., 2008; Salerno et al., 2009; Macqueen et al., 2010a; Mobley et al., 2014; Sun et al., 2016), *Xenopus* neural development (Liu et al., 2017), meiosis/carcinogenesis (Komiya et al., 2008; Macqueen et al., 2010b; Clemons et al., 2013), tick stress response, feeding, growth and reproduction (Almazán et al., 2003, 2005; de la Fuente et al., 2006a, 2008; Smith et al., 2009; Busby et al., 2012; Rahman et al., 2018), pathogen infection and transmission in ticks (de la Fuente et al., 2006b, 2016, 2017a; Zivkovic et al., 2010a,b; Busby et al., 2012; Hajdušek et al., 2013) and turbot (Yang et al., 2011), human glioblastoma cell apoptosis (Krossa et al., 2015), cattle marbling (Sasaki et al., 2009; Watanabe et al., 2011; Kim et al., 2013), and mouse mitogen-induced B cell cycle progression and humoral immune responses (Tartey et al., 2015). For example, as previously reported in *I. scapularis* and other tick species (de la Fuente and Kocan, 2006; de la Fuente et al., 2006a,b, 2011, 2013; Merino et al., 2013a; de la Fuente and Contreras, 2015), Subolesin appears to function in multiple biological processes such as tick response to infection, feeding, reproduction, development and stress response (Figure 6).

**FIGURE 7:**
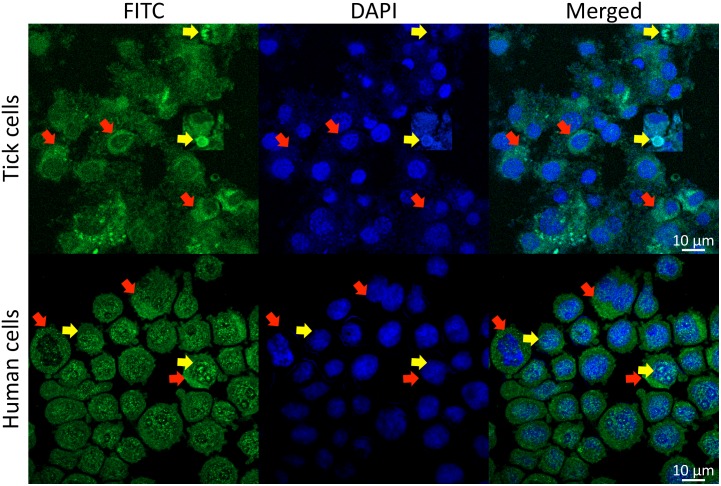
Subcellular localization of Subolesin/Akirin2. Representative images of immunofluorescence analysis of *I. scapularis* ISE6 and human HL60 cells incubated with anti-Subolesin and anti-Akirin2 antibodies, respectively. The cells were fixed with 4% paraformaldehyde in PBS for 15 min, permeabilized with 0.5% Triton X-100 in PBS for 5 min and blocked with blocking buffer (3% BSA in PBS) for 1 h. Then, the cells were incubated overnight at 4°C with anti-Subolesin (Antunes et al., 2014) or anti-Akirin2 (Abcam, Cambridge, United Kingdom) antibodies (1/50 dilution in 3% BSA in PBS). After 3 washes with PBS, the slides were incubated with fluorescein isothiocyanate (FITC) conjugated anti-rabbit secondary antibodies (Sigma-Aldrich, St. Louis, MO, United States; green) at 1/160 dilution in 3% BSA in PBS for 1 h at room temperature. Cells were counterstained with ProLong Antifade containing 4′,6-diamidino-2-phenylindole (DAPI) (Molecular Probes, Eugene, OR, United States; blue), and imaged with a Zeiss LSM800 confocal microscope using a 63× oil immersion lens (Carl Zeiss, Oberkochen, Germany). Yellow arrows show examples of protein localization in the nucleus while red arrows illustrate protein localization in the cytoplasm.

Akirin1 and Akirin2 have also different functions in vertebrates, which is illustrated by the role of Akirin1 in myogenesis while Akirin2 promotes meiosis/carcinogenesis (Macqueen and Johnston, 2009; Macqueen et al., 2010a,b; Figure 4). These different functions may be related to the Akirin subcellular localization. While Akirin1 is found in the nucleus, Subolesin/Akirin2 is located in both cytoplasm and nucleus (de la Fuente et al., 2011; Antunes et al., 2014; Krossa et al., 2015; Pavithiran et al., 2018; Figure 7). The subcellular localization of Subolesin/Akirin2 is probably related to its structure, which as discussed above contains NLS domains that are involved in protein transport in and out of the nucleus via nuclear pores (Leung et al., 2003).

In summary and based on current information, Subolesin/Akirin evolved with similar functions in both invertebrates and vertebrates (Figure 5). The annotation of some biological processes described in certain taxa only may be due to the presence of species-specific functions or more likely a consequence of the still incomplete characterization of Subolesin/Akirin function in the different species.

## Subolesin/Akirin Role in Cell Interactome and Regulome

Subolesin/Akirin are proteins without catalytic or DNA-binding capacity. How Subolesin/Akirin regulate gene expression is unknown but likely involve interactions with proteins with DNA-binding or chromatin-remodeling capacity (de la Fuente et al., 2008; Beutler and Moresco, 2008; Nowak and Baylies, 2012; Nowak et al., 2012; Naranjo et al., 2013). In this way, Subolesin/Akirin link the activities of transcription factors with those of chromatin remodeling complexes to influence gene expression in a context-dependent manner (Nowak and Baylies, 2012; Nowak et al., 2012). For example, Subolesin/Akirin and Akirin2 as key components of the innate immune response can directly or indirectly interact with other regulatory proteins such as “14-3-3” proteins, DNA methyltransferase-associated protein 1 (DMAP1) and the basic helix–loop–helix transcription factor (Twist) to up- or down-regulate transcription (Gonzalez and Baylies, 2005; Goto et al., 2008, 2014; Komiya et al., 2008; de la Fuente et al., 2008; Beutler and Moresco, 2008; Nowak et al., 2012; Naranjo et al., 2013; Tartey et al., 2014; Gulia-Nuss et al., 2016; Shaw et al., 2017).

**FIGURE 8:**
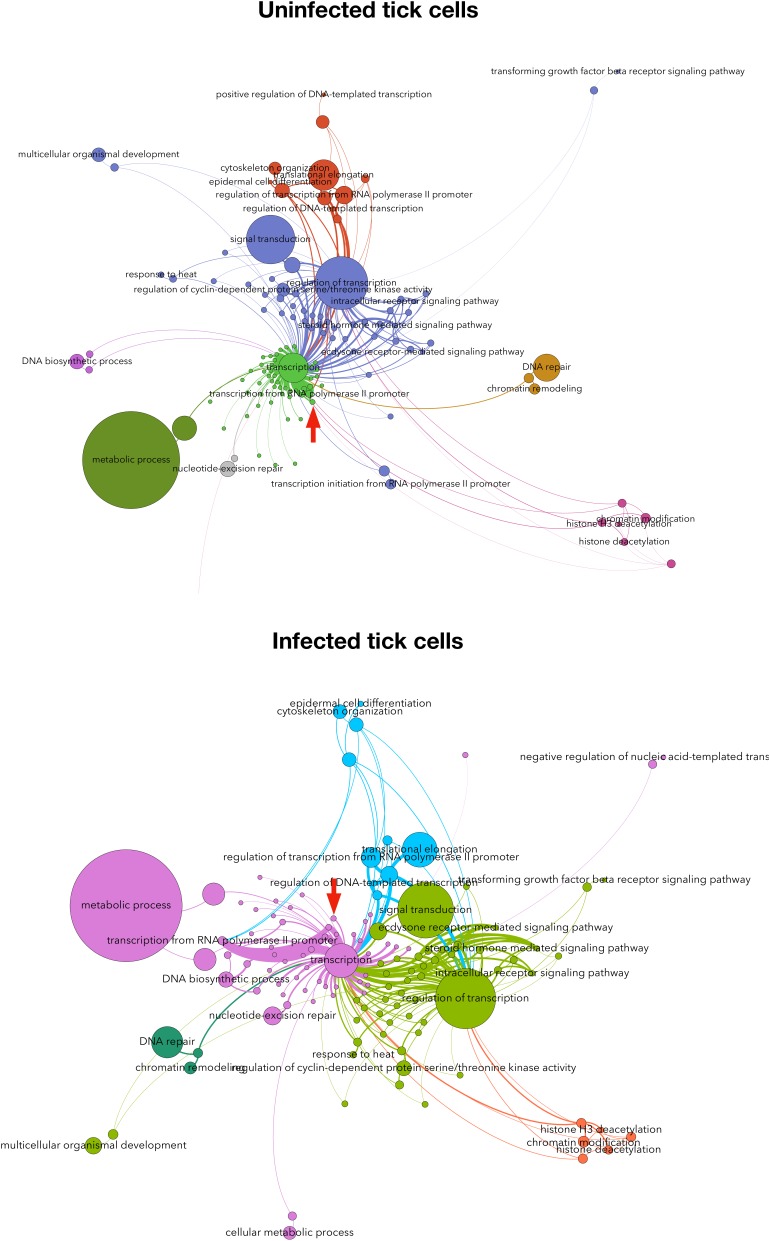
Subolesin regulome in tick cells. The network of proteins and processes associated to transcription in tick cells uninfected and infected with *Anaplasma phagocytophilum*. The nodes (circles) are either proteins or processes (labeled). The size of each circle is proportional to its centrality index. The networks show (clusters of interacting proteins and processes in colors. The width of each link is proportional to the strength of the interaction. The networks show the topology of the tick interactome and regulome. The networks were built with the annotated proteins represented in either uninfected or infected cells, and a directed network was built for each protein linked to the processes in which it is involved. The weight of each link is proportional to the number of reads of the protein. This weighted degree of each link was used to calculate the centrality indexes, mainly the Betweenness Centrality, which is represented in the panels. Only the proteins annotated as involved in processes associated with transcription (i.e., linked by one or more protein(s) simultaneously annotated as transcripiton or other cellular process). The topology of the networks was obtained with the Lovaine algorithm. In both networks, the topological position of Subolesin is marked with a red arrow. Methods were described in Estrada-Peña et al. (2018).)

The Subolesin/Akirin role in the cell interactome and regulome in response to different stimuli has not been characterized. Recently, we proposed a method based on the graph theory for the analysis of human and tick cell proteome in response to *A. phagocytophilum* infection (Estrada-Peña et al., 2018). This approach resulted in a network of interacting proteins and cell processes clustered in biological pathways, and ranked with indexes representing the topology of the proteome influenced by features of the interactome and regulome. The results evidenced differences in the response to *A. phagocytophilum* infection between human and tick cells, and supported that human neutrophils but not tick cells limit pathogen infection through differential representation of ras-related proteins (Estrada-Peña et al., 2018). Herein, this method was applied to predict the position of Subolesin in the regulome of tick cells and in response to *A. phagocytophilum* infection (Figure 8). The results showed that Subolesin is deeply involved in the core of transcription processes but also in other secondary processes such as transcription from RNA polymerase II promoter, DNA repair, and chromatin remodeling (Figure 8). Furthermore, other processes that change in infected cells when compared to uninfected cells (i.e., signal transduction, regulation of transcription, and response to heat) are deeply linked to the central transcription process. The putative Subolesin role in these processes varied between infected and uninfected cells (the width of the lines is proportional to the importance of the link between proteins and processes; Figure 8). For example, it appears that particularly in infected cells other proteins but Subolesin have a more prominent role in the strong protein link with transcription and transcription from RNA polymerase II promoter processes (Figure 8). These results predict the role that Subolesin plays in the regulation of different biological processes, and its differential role in response to *A. phagocytophilum* infection in tick cells. However, the lack of a more prominent role for Subolesin may respond to the fact that this protein does not bind directly to DNA but interacts with other proteins to exert its regulatory function.

**FIGURE 9:**
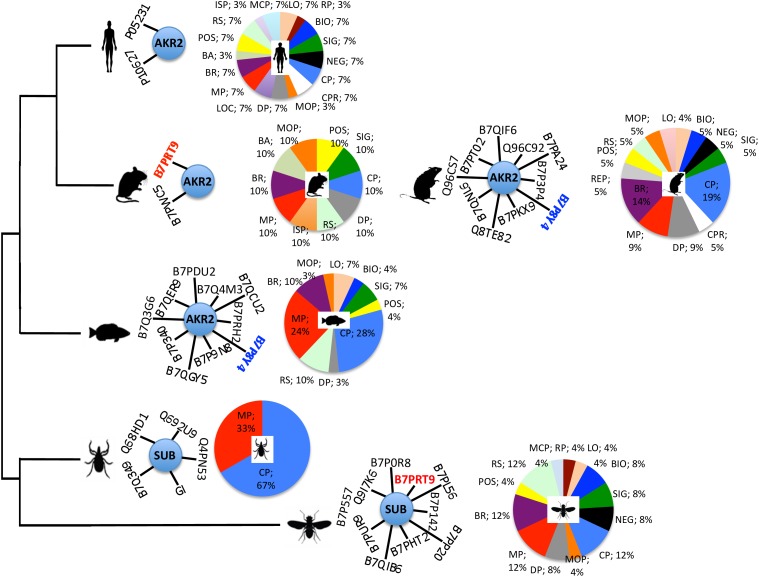
Characterization of the Subolesin/Akirin2 interactome. The information on Subolesin/Akirin-protein physical and functional interactions was compiled from the String protein-protein interactions database v.10.5 (https://string-db.org). The central node of the networks represent Subolesin/Akirin2 while the edges correspond to the predicted functional associations. Only predictions with medium (or better) confidence ( > 0.4) limited to the top 10 interactions with protein-protein interaction (PPI) enrichment *p*-value ≤ 0.5 were considered. To compare the different species, protein annotations were standardize by identity to *I. scapularis/I. ricinus*-*D. melanogaster*-*H. sapiens* order of priority (see Supplementary Dataset S1 for complete annotations). For illustration purposes, the species included in the analysis correspond to *D. melanogaster, I. scapularis, Danio rerio, Mus musculus, Rattus norvegicus*, and *H. sapiens*. Identical proteins in two different species are highlighted in red and blue letters. The functional annotation of the Subolesin/Akirin2 interacting proteins according to the biological processes (level 2) in which they are involved was done using Blast2GO (www.blast2go.com), and represented in pies with different colors for each process and the percentage of proteins on each process. Abbreviations: LO, localization (sepia); RP, rhythmic process (sangria); BIO, biogenesis (blue); SIG, signaling (green); NEG, negative regulation of biological process (black); CP, cellular process (azure); CPR, cell proliferation (white); MCP, multi-organism process (sky); DP, developmental process (gray); LOC, locomotion (violet); MP, metabolic process (red); BR, biological regulation (byzantine); BA, biological adhesion (moss); POS, positive regulation of biological process (yellow); RS, response to stimulus (tea); ISP, immune system process (gold); MOP, multicellular organismal process (orange); REP, reproductive process (smoke). Color code was established according to color thesaurus (https://graf1x.com/list-of-colors-with-color-names/).

In an attempt to provide information on the Subolesin/Akirin interactome, the information on Subolesin/Akirin-protein physical and functional interactions was compiled from the String protein-protein interactions database^2^ (Figure 9 and Supplementary Dataset S1). Based on the analysis of protein-protein interactions, the results did not allow establishing an evolutionary signature of the Subolesin/Akirin2 interactome (Figure 9), probably due to the limited information available. Nevertheless, similar Subolesin/Akirin2 interacting proteins were described in fly and mouse (B7PRT9, Brahma/SWI2-related protein BRG-1) and in fish and rat (B7P8Y4, Arginyl-tRNA synthetase) suggesting possible evolutionarily conserved protein-protein interactions (Figure 9). To further gain insight into the evolution of the Subolesin/Akirin2 interactome, instead of looking only at protein-protein interactions, the interacting proteins were annotated according to the biological processes in which they are involved (Figure 9). The results of this analysis showed that the biological processes affected by the Subolesin/Akirin interactome are evolutionarily conserved, with metabolic process (MP), cellular process (CP) and biological regulation (BR) being among the most represented processes in all organisms (Figure 9).

## Protective Capacity of Subolesin/Akirin for the Control of Ectoparasite Vector Infestations and Pathogen Infection

Subolesin was discovered and characterized as a tick protective antigen for the control of *I. scapularis* infestations (Almazán et al., 2003, 2005; Sonenshine et al., 2006). Since then, Subolesin/Akirin showed a protective capacity in vaccines for the control of infestations by different arthropod species and pathogen infection and transmission (reviewed by de la Fuente et al., 2006a, 2011, 2013; de la Fuente and Kocan, 2006, 2014; Merino et al., 2013a,b; de la Fuente and Contreras, 2015). The putative mechanism for Subolesin vaccine protection was described by de la Fuente et al. (2011). They showed that by still unknown mechanisms anti-Subolesin antibodies could enter into tick cells where they can interact with cytosolic Subolesin to prevent its translocation to the nucleus and therefore the possibility to exert it regulatory functions.

**FIGURE 10:**
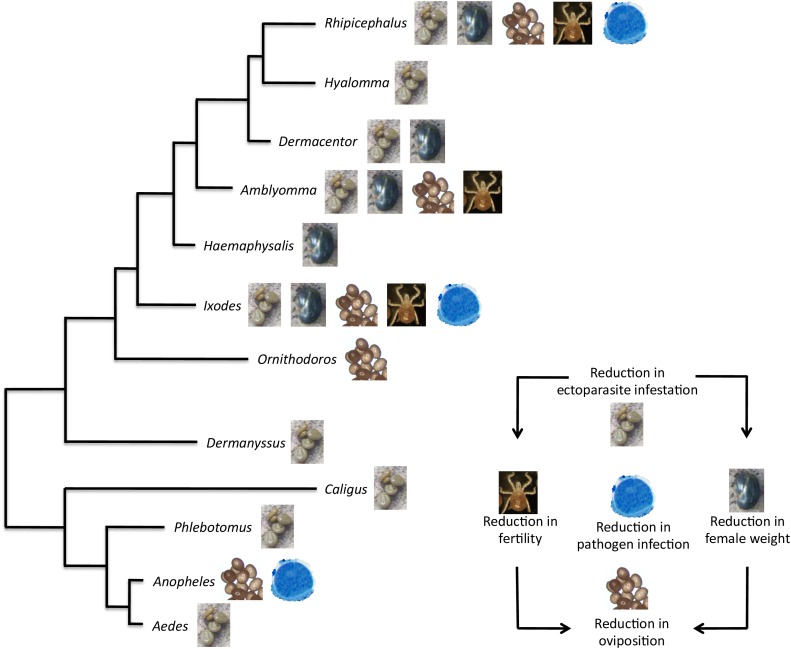
Protective capacity of Subolesin/Akirin in vaccines for the control of arthropod ectoparasite infestations and pathogen infection. The effect of Subolesin/Akirin vaccination is shown on evolutionarily diverse arthropod genera. The effect of the vaccine was recorded on different phases of ectoparasite life cycle in the form of reduction in ectoparasite infestation (number of ectoparasites completing feeding), weight (weight of engorged female ectoparasites), oviposition (number of eggs per female), and fertility (number of larvae per female) in ectoparasites fed on vaccinated hosts when compared to controls. The reduction in pathogen infection was recorded as differences in pathogen levels between ectoparasites fed on vaccinated and control hosts.

The development of vaccines for the control of multiple arthropod ectoparasites constitutes a priority for targeting the various species infesting the same host (de la Fuente et al., 2011; Contreras and de la Fuente, 2016b). In this context, Subolesin/Akirin appears as a promising vaccine protective antigen due to its conservation in sequence and function through evolution (Figures 1, 5, 9). In fact, recent results support the potential of Subolesin/Akirin as a vaccine protective antigen for the control of multiple ectoparasite vector species and transmitted pathogens (Figure 10). The arthropod ectoparasite species in which Subolesin/Akirin have shown protective capacity by affecting different phases of their life cycles include the genera *Aedes, Anopheles, Phlebotomus, Caligus, Dermanyssus, Ornithodoros, Ixodes, Haemaphysalis, Amblyomma, Dermacentor, Hyalomma* and *Rhipicephalus* (Almazán et al., 2003, 2005, 2010, 2012; de la Fuente et al., 2006a, 2010, 2011, 2013; Canales et al., 2009; Harrington et al., 2009; Zivkovic et al., 2010a; Moreno-Cid et al., 2011, 2013; Carpio et al., 2011; Bensaci et al., 2012; Merino et al., 2011a,b, 2013b; Carreón et al., 2012; Manzano-Román et al., 2012, 2015; de la Fuente and Kocan, 2014; da Costa et al., 2014; Shakya et al., 2014; Torina et al., 2014; Contreras et al., 2015; de la Fuente and Contreras, 2015; Lu et al., 2016; Olds et al., 2016; Contreras and de la Fuente, 2016a; Kumar et al., 2017; Villar et al., 2017; Figure 10). The reduction in pathogen infection has been shown for tick-borne pathogens, *Anaplasma marginale, A. phagocytophilum, Borrelia burgdorferi* s.s. and *Babesia bovis*, and the mosquito-borne pathogen, *Plasmodium berghei* (de la Fuente et al., 2011; Merino et al., 2011b, 2013a; Bensaci et al., 2012; de la Fuente and Kocan, 2014; da Costa et al., 2014; Figure 10). Furthermore, protective epitopes were mapped in Subolesin/Akirin (Prudencio et al., 2010) and chimeric antigens were designed showing protective capacity in vaccinated hosts against tick infestations (Merino et al., 2013b; Contreras and de la Fuente, 2016a).

Considering the protective capacity shown by Subolesin/Akirin on different vector and pathogen species, future research directions will include the characterization of its protective capacity in other arthropod ectoparasite species, and the combination with other vector-derived and pathogen-derived antigens to increase vaccine efficacy for the control of both vector infestations and pathogen infection (Schetters et al., 2016; de la Fuente et al., 2017b; de la Fuente, 2018).

## Conclusion and Future Directions

Significant advances have been made recently toward understanding the evolution and function of Subolesin/Akirin. Our results suggest that Subolesin/Akirin evolved conserving not only its sequence and structure, but also its function and role in cell interactome and regulome in response to pathogen infection and other biological processes. However, major challenges remain in fully characterizing the interactome and function of these proteins, their role in the cell regulome in response to different stimuli, and how their evolution can meet species-specific demands. Furthermore, the structure of Subolesin/Akirin and interacting molecules should be resolved by X-ray crystallography to better understand their function. Finally, the conserved functional evolution of Subolesin/Akirin correlates with the protective capacity shown by these proteins in vaccine formulations for the control of different arthropod and pathogen species, and encourage further research to develop new vaccine formulations by combining Subolesin/Akirin with interacting proteins for the control of multiple ectoparasite infestations and pathogen infection.

## Author Contributions

JdlF conceived the paper. SA-J, MV, AC-C, JV, and AE-P performed the data analyses. PA and SA-J performed the microscopy studies. JF, SA-J, JV, and AC-C wrote the manuscript. All authors approved and contributed to the final version of the manuscript.

## Conflict of Interest Statement

The authors declare that the research was conducted in the absence of any commercial or financial relationships that could be construed as a potential conflict of interest.

## Footnotes

1 http://predictioncenter.org

2 https://string-db.org

## Supplementary Material

FIGURE S1 Phylogenetic tree of akirin and subolesin nucleotide sequences. The figure displays a Neighbor Joining (NJ) phylogenetic tree of 361 nucleotide sequences belonging to 152 families, 73 orders and 15 classes. GenBank accession numbers and species names are shown. Tree reconstruction method was as described in Figure 1.

DATASET S1 Annotation of the Subolesin/Akirin interactome. The information on Subolesin/Akirin-protein interactions was compiled from the literature and String protein-protein interactions database v.10.5 (https://string-db.org). Only predictions with medium (or better) confidence (0.4) limited to the top 10 interactions with protein-protein interaction (PPI) enrichment *p*-value ≤ 0.5 were considered. To compare the different species, protein annotations were standardize by identity to *I. scapularis/I. ricinus*-*D. melanogaster*-*H. sapiens* order of priority.
