# Supplementary figures and images for: Functional Evolution of Subolesin/Akirin
Source: Front Physiol. 2018 Nov 13;9:1612. doi: 10.3389/fphys.2018.01612 (PMC6277881; doi:10.3389/fphys.2018.01612)

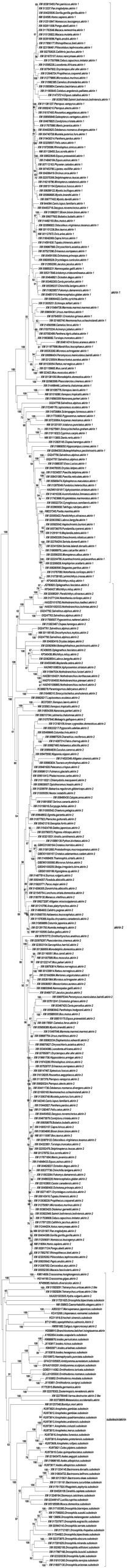

Supplement: FIGURE S1 — Phylogenetic tree of akirin and subolesin nucleotide sequences. The figure displays a Neighbor Joining (NJ) phylogenetic tree of 361 nucleotide sequences belonging to 152 families, 73 orders and 15 classes. GenBank accession numbers and species names are shown. Tree reconstruction method was as described in Figure 1. [file Image_1.pdf]
